# Exploring the Use of Meditation as a Valuable Tool to Counteract Sedentariness

**DOI:** 10.3389/fpsyg.2020.00299

**Published:** 2020-02-25

**Authors:** Marcelo Bigliassi, Romulo Bertuzzi

**Affiliations:** School of Physical Education and Sport, University of São Paulo, São Paulo, Brazil

**Keywords:** applied psychology, attention, exercise, meditation, mindfulness, physical activity

## Abstract

Some forms of meditation have been recently proposed as effective tools to facilitate the handling of undesired thoughts and reappraisal of negative emotions that commonly arise during exercise-related situations. The effects of meditation-based interventions on psychological responses could also be used as a means by which to increase exercise adherence and counteract the detrimental consequences of sedentariness. In the present article, we briefly describe the effects of meditation on physical activity and related factors. We also propose a theoretical model as a means by which to further understanding of the effects of meditation on psychological, psychophysical, and psychophysiological responses during exercise. The results of very recent studies in the realms of cognitive and affective psychology are promising. The putative psychological mechanisms underlying the effects of meditation on exercise appear to be associated with the interpretation of interoceptive and exteroceptive sensory signals. This is primarily due to the fact that meditation influences the cerebral processing of physical sensations, emotions, and thoughts. In such instances, the bodily and perceptual responses that are commonly reported during exercise might be assuaged during the practice of meditation. It also appears that conscious presence and self-compassion function as an emotional backdrop against which more complex behaviors can be forged. In such instances, re-engagement to physical activity programs can be more effectively achieved through the implementation of holistic methods to treat the body and mind. The comments provided in the present paper might have very important implications for exercise adherence and the treatment of hypokinetic diseases.

## Introduction

Sedentariness has grown exponentially and some of the recently proposed interventions to counteract physical inactivity appear to be inefficacious (cf. McDermott et al., 2018). In the United Kingdom, for example, more than 20 million adults are classified as physically inactive, what consequently increases the risk of cardiovascular complications and costs the government ∼1.2 billion sterling pounds each year (Townsend et al., 2015). Sedentary behavior has also been associated with poor mental health by numerous studies (Hoare et al., 2016; Ellingson et al., 2018). This association can be accounted for by the fact that sedentary individuals are usually more likely to experience shame, self-criticism, and perceptions of inferiority when compared to people who are physically active (Chastin et al., 2014; Castonguay et al., 2015; Gaston et al., 2016). In actuality, an almost linear relationship has been proposed between negative psychological traits (e.g., anxiety and depression) and sedentary behavior (see Pedersen and Saltin, 2015). Over the past decade, it has become undeniable that physical and mental health are utterly entangled (Kangasniemi et al., 2014). More recently, it has been suggested that some psychological factors are not only associated with the frequency at which individuals exercise but they could actually mediate the perception-action cycle (i.e., it could hamper/facilitate execution of movements; Demarzo et al., 2014).

Despite recent advances in science and technology such as the use of mobile applications to monitor physiological parameters during exercise (e.g., heart rate), sedentariness continues to rise at an alarming rate. The reasons behind this phenomenon appear to be due primarily to a lack of psychological skills such as emotional stability, ability to focus, compassion, and gratitude (Stubbs et al., 2018). Unsurprisingly, compelling evidence indicates that conscious presence and self-compassion have the potential to *mediate* mental and physical health (Dunne et al., 2018). Therefore, it appears logical to postulate that sedentary individuals might need to relearn how to control their thoughts and feelings as a means by which to create an emotionally stable foundation on which behavior can be altered (Hogan et al., 2015). In this article, we will briefly discuss the effects of meditation techniques as a means by which to reconnect with our senses and enhance self-compassion with the ultimate goal of increasing physical activity. We will also propose a series of methods (e.g., body scan and breathing meditation) that can be used during various exercise modalities in order to make a given activity more pleasurable and consequently enhance affective responses (Jislin-Goldberg et al., 2012). Such methods also have the potential to facilitate execution of movements, assuage fatigue-related sensations such as breathlessness and limb discomfort, and increase exercise adherence (Birrer et al., 2012).

It is noteworthy that although meditation-based interventions have been only recently proposed in the fields of physical activity and public health (Birrer et al., 2012), they have been utilized quite extensively in other realms of knowledge such as clinical psychology, behavioral medicine, and psychiatry (Körner et al., 2015; Janssen et al., 2018). Meditation-based interventions can also be used by people who are physically active (see e.g., Jones and Parker, 2016; Colzato and Kibele, 2017) in order to optimize the neural control of working muscles and elicit more positive affective responses during exercise- and sport-related tasks. Accordingly, we have also explored some psychological mechanisms that could potentially underlie the effects of meditation on exercise tasks performed at various intensities. Finally, we have proposed a series of practical interventions that could be tested by researchers and exercise professionals as valuable tools to facilitate reappraisal of internal bodily sensations during execution of movements.

## Why Meditation Could Help

It has been frequently demonstrated that physically inactive individuals are generally more susceptible to psychological distress, anxiety, and depression (e.g., De Mello et al., 2013; Stonerock et al., 2015; Stubbs et al., 2018). Researchers and exercise professionals are also aware that by re-engaging in physical activity programs, sedentary individuals tend to experience more positive emotional responses and amelioration of anxiety (e.g., Hogan et al., 2015; Biddle, 2016; Ellingson et al., 2018). However, negative psychological states may have a detrimental effect on exercise re-engagement (Nasstasia et al., 2018; Averill et al., 2019). In other words, individuals who constantly experience poor mental health might find it particularly difficult to become active. This is mainly because re-engagement in physical activity programs usually requires certain levels of motivation, self-discipline, and willpower, and such psychological abilities can be partially compromised in sedentary individuals (Brand and Ekkekakis, 2018; Castonguay et al., 2018). In such instances, it appears logical to surmise that tackling the psychological traits through the use of objective and practical interventions might be an interesting approach to re-construct a solid foundation on which physical activity behavior can be altered. Therefore, we propose the use of meditation-based interventions as a valuable tool to counteract sedentariness through the promotion of well-being and improvement of psychological functioning (Janssen et al., 2018; Lacaille et al., 2018). Meditation-based interventions may hold the potential to make people more willing to act compassionately (Condon et al., 2013), leading to a series of psychological events such as enhanced self-awareness. This sequence of psychological reactions is hypothesized to stimulate self-care and preservation through the implementation of healthy behaviors (Callaghan, 2003).

### Types of Meditation

The earliest records of meditation practice indicate that this form of attentional manipulation/observation originated in India thousands of years ago (Wynne, 2007). There are two main types of meditation and a wide variety of components and elements that can be implemented such as mantras and physical exercises. The first type of meditation is named “focused attention” and refers to the allocation of attentional focus to a single object (e.g., breathing pattern, a mantra, and external object). During the practice of focused attention meditation individuals are commonly guided by someone such as an instructor who functions as a guide to facilitate the reallocation of attention to the target and prevent distractions from entering focal awareness (Ainsworth et al., 2013; Lippelt et al., 2014). Zazen, Samatha, Kundalini, Pranayama, and Qigong are common variations of focused attention meditation; each of which with its peculiarities. For example, counting the breath is commonly used as one of the main techniques to improve attentional control during the practice of Zazen meditation. Conversely, Qigong’s meditation routines usually involve the use of movements (e.g., stretching) in combination with visualization and breathing exercises to stimulate concentration. It is also noteworthy that Western countries commonly refer to some of these meditation styles as breathing meditation, body scan, Zen meditation, etc.

The second type of meditation is commonly referred to as “open monitoring” or “non-directive,” which basically means that individuals are not required to direct their attentional foci to a single object; in this case, they are simply required to consciously monitor all aspects of their experiences such as thoughts, physical sensations, and memories (Colzato and Kibele, 2017). Another important aspect related to the practice of open monitoring meditation is the fact that participants are frequently reminded to not judge their thoughts, memories, and feelings. In such instances, they are asked to simply observe whatever they experience without judgment or attachment, as if they could position themselves as a third person outside their bodies and merely witness how their thoughts and emotions *move* and *transform* over time. Vipassana is one of the most common forms of open monitoring meditation, where participants are usually asked to observe their breath and notice how the physical sensations and emotional responses change throughout the session. Another interesting type of open monitoring meditation is named Taoist, which uses a peculiar technique of visualization in combination with breathing exercises as a form of *healing process* (e.g., “As you breathe out, allow all the pain and stress to leave your body”). It is worth noting that the types of meditation described in this subsection are just examples and do not represent the practice of meditation in its entirety. There might be hundreds, if not thousands of meditation routines, including all the possible elements and combinations. Therefore, the present authors simply intended to provide the readers with some of the most seminal foundations and elements of the practice of meditation.

It is also important to emphasize that although some meditation routines have been extensively investigated by the scientific community (e.g., mindfulness and transcendental meditation; Lacaille et al., 2018; Munoz et al., 2018), others still need to be further explored. Thus, comparing the efficacy and effectiveness of various types of meditation might require a more substantial number of controlled trials. Recent studies have also identified that certain meditation techniques may be more appropriate for specific situations (Hildebrandt et al., 2017; Trautwein et al., 2019), and that the effects of meditation are moderated by a wide range of personal, environmental, and situational factors. It is logical, therefore, that such factors may also influence the effects of meditation on exercise. The main objective of this article is to simply identify, based on the main characteristics of some meditation styles, what would be the most interesting aspects to consider during exercise-related situations. Moreover, exercise conditions also differ substantially in terms of complexity, intensity, modality, and duration. Accordingly, it is reasonable to assume that each exercise condition might require specific meditation routines.

### Mindfulness-Based Interventions to Increase Physical Activity

The main difference between the concepts of “meditation” and “mindfulness” is that the latter refers to the act of focusing on being in the present moment without judgment (e.g., focus on the taste, texture, and smell of food), whereas meditation is the umbrella term and refers to the practice of reaching consciousness (e.g., through the use of mindfulness-based interventions or other forms of meditation). During the practice of mindfulness, interoceptive (e.g., breathing) and exteroceptive (e.g., auditory signals) cues are commonly used as sources of information to focus on. It is also important to emphasize that the mind *wanders*, and individuals are commonly asked to not judge their thoughts and gently bring their attentional focus back to the target. The practice can vary from a couple of minutes to many hours and can be conducted literally at any place (e.g., gymnasia and health centers). The final goal is generally to remind ourselves that the past no longer exists, and the future is unpredictable. In other words, the present moment is all that we actually *have*. The practice of mindfulness is grounded on the premise that the constant reallocation of attention toward the present moment can lead to residual effects that will pervade during other activities of daily life. This form of meditation session appears to have a significant effect on anxiety-related symptoms, emotional stress, and cognition (Ainsworth et al., 2013; van der Zwan et al., 2015).

In a recent study conducted by Tsafou et al. (2016) the authors explored the relationship among mindfulness, physical activity, and perceived satisfactions in a sample comprised of 398 participants. The findings of this study indicated that participants who were mindful about their movements during physical activity were also more likely to engage in exercise programs. The authors have also identified that the relationship between mindfulness and physical activity engagement appears to be mediated by how satisfied participants felt during the execution of movements. Along similar lines, other studies have indicated that mindfulness have the potential to facilitate awareness of positive emotions, leading to subsequent psychobiological, psychophysical, and behavioral outcomes (Erisman and Roemer, 2010; Jislin-Goldberg et al., 2012). In such instances, it appears reasonable to assume that awareness of positive emotions experienced during exercise could potentially facilitate the handling of undesired thoughts that commonly arise in response to fatigue-related sensations (Parry et al., 2011). Therefore, mindfulness-based interventions can also be used during the most critical periods of the exercise regimen as a means by which to partially inhibit negative bodily sensations, elicit a more positive affective experience, and improve exercise behavior (Kang et al., 2017).

Researchers have also used mindfulness-based interventions as a means to facilitate weight-loss and improve health-related behaviors in adults with overweight and obesity. The results of a systematic review and meta-analysis recently published by Ruffault et al. (2017) confirm that mindfulness training (i.e., the repetitive process of shifting attention to the present moment) is effective in reducing impulsive eating and increasing physical activity among adults with excess body fat. The authors hypothesized that the underlying mechanisms could be associated with the fact that mindfulness-based interventions have residual effects after cessation of the practice that could naturally force individuals to be more mindful about other aspects of life such as exercise behavior and eating habits (cf. Daubenmier et al., 2016).

Rosenstreich et al. (2018) have also investigated the associations between facets of mindfulness (i.e., awareness, non-reacting, non-judging, observing, and describe), attention deficit, and postural stability. The authors assessed task performance by measuring participants’ mediolateral trunk sway, and identified, through the use of regression analysis, a positive association between static balance and the facet of observing (i.e., a core aspect of mindfulness; Lilja et al., 2013) in the absence of visual cues. Interestingly, other facets of mindfulness were associated with less stable performance when participants reported divided attention during execution of the motor task. The complexity of the psychological underpinnings of mindfulness revealed by Rosenstreich et al. (2018) also serve to indicate that mindfulness-based interventions hold the potential to hamper or facilitate execution of movements, and that specific recommendations must be proposed on the basis of their efficacy and functionality. This study also demonstrates that the use of certain forms of meditation might be associated with decrements in motor performance. In actuality, the constant reallocation of attentional focus toward the present moment without proper instruction can potentially lead to an increase in the use of associative thoughts and perceptions of negative thoughts and emotions. In such instances, it appears logical that individuals with no experience in meditation should receive specific recommendations on how to carefully and progressively implement the practice of meditation as a daily routine.

The use of mindfulness-based interventions may also compromise execution of certain movement patterns and potentially elicit more negative affective responses. This is mainly due to the fact that an internal focus of attention is generally associated with poor motor performance during execution of a wide range of physical tasks (for review, see Wulf, 2013). An increase in the use of associative thoughts can make individuals overly conscious of their movement patterns, which may naturally force the interpretation of task-related factors and interoceptive information (cf. Lohse and Sherwood, 2012) and elicit feelings of displeasure (Rejeski, 1985; Rose and Parfitt, 2010). This is the main reason why mindfulness-based interventions should be carefully administered as a means by which to aid in the reappraisal of one’s emotional state (Noetel et al., 2017), potentially leading to a phenomenon where internal sensory cues are perceived with greater acceptance. The present authors hypothesize that such psychophysical processes might lead to a more efficient control of working muscles and subsequent amelioration of fatigue-related symptoms.

It has also become clear that the scientific evidence available to date in this particular topic is very scarce. The use of mindfulness training in the field of physical activity has been only seldom investigated (Kang et al., 2017; Bigliassi et al., 2020), but the results from adjacent domains such as cognitive psychology and behavioral medicine suggest that long-term interventions are highly successful (Daubenmier et al., 2016; Janssen et al., 2018). Future research is still necessary to elucidate the acute effects of mindfulness-based interventions on affective responses (e.g., perceived enjoyment), fatigue-related symptoms (e.g., breathlessness and limb discomfort), arousal regulation, and task performance. It is also necessary to further our understanding of the brain mechanisms that underlie such positive effects and explore the interaction between central nervous system and peripheral physiological reactions (e.g., changes in heart rate variability) during and after the practice of mindful physical activity.

### Self-Compassion and Exercise Behavior

On a daily basis, many individuals wonder why they should reengage in physical activity programs (O’Hara et al., 2014). This type of question is, sometimes, very powerful and commonly *sticks* in one’s mind for months or years. Over time, our bodies get used to the lack of movement and we adapt slowly to this new behavioral pattern (i.e., sedentariness). Eventually, physically inactive/sedentary individuals feel the need to exercise, but psychosocial factors (e.g., shame and guilt) and psychophysical responses (e.g., extreme heaviness in the muscles) are too challenging to deal with (Sabiston et al., 2010). Breaking this cycle simply becomes very difficult and everything appears to be a huge obstacle. Interestingly, compelling evidence indicates that lack of physical activity appears to be directly linked with one’s inability to love her/himself (Hall et al., 2013). This is due to the fact that self-compassion is a basic premise for more complex actions such as exercise behavior. For example, Magnus et al. (2010) identified that self-compassion is associated with well-being in the exercise context and suggested the possibility that the development of self-compassion might somehow facilitate adherence to physical activity programs. In such instances, self-compassion assumes the position of an emotional requirement and has the propensity to influence both mental and physical health (Dunne et al., 2018).

It has also been hypothesized that we partially lost the ability to love ourselves the same way we forgot how to be present (Hall et al., 2013; Körner et al., 2015), meaning that self-compassion needs to be trained the same way we use mindfulness-based interventions to up-/down-regulate psychophysiological arousal [i.e., activation levels (how worked-up one feels)]. For example, Semenchuk et al. (2018) recently investigated the relationship between self-compassion and exercise self-regulation. The authors identified that self-compassion was positively associated with self-determined motivation and exercise goal re-engagement, and negatively related to extrinsic motivation, state rumination, and negative affective responses. Accordingly, techniques to develop self-compassion can be used as valuable tools to assuage negative emotions and facilitate re-engagement in physical activities.

There are numerous ways of learning how to be more compassionate. In this review article, we briefly delineate some of the methods related to the use of meditation techniques to enhance self-compassion. As part of the process to learn on how to develop self-compassion, individuals need to explore the true meaning of suffering and understand that suffering is part of life (i.e., we are not alone; Smeets et al., 2014). Commonly applied interventions require participants to repeat sentences in their minds (e.g., “Other people feel the same way I feel”; see Table 1), so the meaning of the words can resonate and create a background on which self-compassion can be developed (Körner et al., 2015). Subsequently, individuals are asked to continue the same exercise, but at this time, replacing the sentences with phrases of compassion toward themselves (e.g., “May I learn to accept myself as I am”).

**TABLE 1:** Practical examples provided for a series of meditation techniques and exercise intensities.

Meditation techniques	Practical examples	Ventilatory threshold
Breathing meditation	Focus on your breathing. The air that moves in and out as you breathe is the fuel to exercise. Do not try to change your breathing pattern in any way. Your body knows exactly how much air it needs.	Below
	Focus on the feeling of air that constantly moves through your nose as you breathe in and out. Remember that the air will provide your muscles with sufficient oxygen to persevere.	Above
Body scan meditation	Bring your attention down to your legs/arms. Try to focus on the physical sensations of your legs/arms. Your body knows exactly how to execute each movement with precision.	Below
	Gently bring your attention to the sensations of discomfort. Your body knows how to fight against the pain you feel. Embrace any pain or discomfort that you are experiencing.	Above
Zen meditation	Observe your thoughts and how they change during the exercise without judgment. Try to focus on all that you can sense, including the environment that surrounds you.	Below
	Do not try to control your thoughts. If you think or feel something, simply accept and embrace it. During the recovery periods try to reflect on how this thought influences your exercise session.	Above
Self-compassion meditation	May I be free from suffering and pain. May I have joy and happiness, not only today but every day from now on. May I accept and respect the physical limitations of my body.	Below
	May I safely endure the pain and discomfort that I experience during this exercise session. May I be strong. May I learn to protect myself against injuries and accidents.	Above
Gratitude meditation	I am grateful for everything I have, including my heart and lungs that allow me to exercise. I am grateful for every second of every day that I get to spend walking/running/cycling.	Below
	I am thankful for the health benefits that this exercise provides. I am grateful for all the friends I make because of the time I spend in the gymnasium/health center.	Above

*The types of meditation and practical examples provided in this table only serve as a sample and do not represent the practice of meditation in its entirety.*

It is very common for individuals to laugh and despise the intervention during the first time they repeat these words. This is because the idea they may have about themselves is opposite to what has been said. However, persistence is key to changing thoughts and behaviors, and this can also be implemented during the execution of gross movements (e.g., while walking or cycling). Unfortunately, the use of self-compassion interventions as a means by which to increase adherence to physical activity programs and enhance affective responses during exercise is currently under-examined. Researchers and exercise professionals are encouraged to explore the use of self-compassion interventions prior to, during, and immediately after exercise sessions. Meditation techniques that are primarily focused on the enhancement of self-compassion are highly likely to elicit positive emotional responses with consequent residual effects on exercise behavior and adherence.

## Putative Psychological and Psychosocial Mechanisms

**FIGURE 1:**
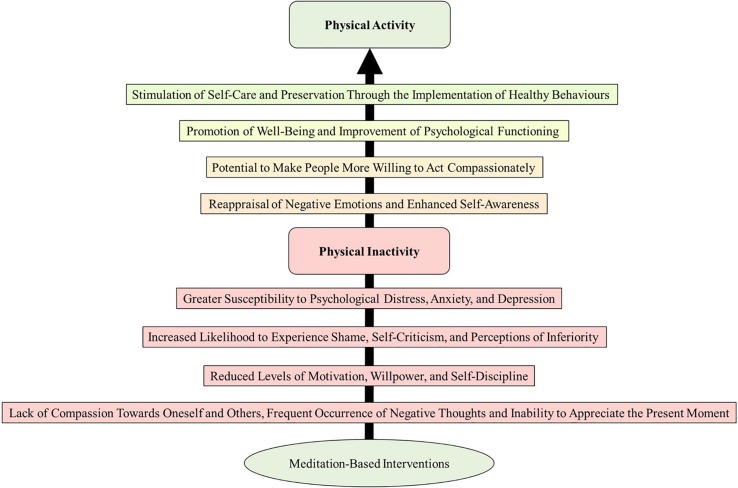
Putative psychosocial mechanisms underlying the effects of meditation-based interventions on exercise re-engagement. The model indicates that the practice of meditation might be particularly effective as a strategy to facilitate emotional reappraisal through the constant reallocation of attention toward the present moment and the repetition of positive messages toward oneself and others. Subsequently, a sequence of psychosocial responses may ultimately stimulate self-care and preservation through the implementation of healthy behaviors.

In this article, we have also explored some of the putative psychological and psychosocial mechanisms underlying the effects of meditation on physical activity behavior. Subsequently, we proposed a theoretical framework (Figure 1) to further understanding of how the practice of meditation can facilitate engagement in physical activity programs. By reviewing the literature, the present authors could observe that reducing sedentariness can be a complex mission that requires a Herculean effort from health professionals (Yang et al., 2019). Researchers and exercise professionals should not expect physically inactive individuals to become active by simply reciting the benefits of regular physical activity. This strategy generally neglects their struggles and may be ineffective in the long-term (see Aunger et al., 2018). Sedentary individuals commonly face difficulties to re-engage in programs of physical activity because of complex and multifaceted psychosocial phenomena (e.g., shame, self-criticism, and perceptions of inferiority). Meditation-based interventions can be used as a means by which to counteract sedentariness through the promotion of well-being and improvement of psychological functioning (Lacaille et al., 2018). Through the continuous reallocation of attention toward the present moment without judgment and the repetition of positive messages toward oneself, negative thoughts and emotions can be perceived with greater acceptance (Keng et al., 2011; Wersebe et al., 2018). Such effects can also be identified in terms of psychophysiological arousal (i.e., experience meditators tend to report significant down-regulations of perceived activation; see Ekici et al., 2018). Consequently, individuals might become more able to experience current physical sensations and positive emotions when their negative thoughts are reappraised (cf. Troy et al., 2018; Katana et al., 2019). In the long term, this sequence of psychological effects is hypothesized to stimulate self-care through the implementation of healthy behaviors, increase motivation and willpower, and facilitate self-discipline (Callaghan, 2003). Accordingly, modulation of such psychological abilities might serve primarily as a form of stimulation toward a more active behavior.

## Meditation-Based Interventions for Physically Active Individuals

Meditation-based interventions may also be used for physically active individuals as a means to optimize the neural control of the musculature, elicit a more positive affective state, and assuage fatigue-related sensations. The putative mechanisms underlying the effects of meditation on exercise appear to be directly associated with the interpretation of interoceptive and exteroceptive sensory signals (e.g., afferent feedback and auditory cues, respectively; Demarzo et al., 2014). This is primarily due to the fact that meditation influences the cerebral processing of physical sensations, emotions, and thoughts (Xu et al., 2014). In such instances, the bodily and perceptual responses that are commonly reported during exercise (e.g., muscle ache and rapid breathing) might be ameliorated during the practice of meditation (Sharon et al., 2016). Meditation routines have the potential to reallocate attentional focus toward environmental sensory signals and reduce processing of internal bodily sensations during light and light-to-moderate exercise intensities. This attentional response serves to prevent afferent feedback from entering focal awareness and protects the central motor command against the detrimental effects of fatigue-related symptoms (Rejeski, 1985). Therefore, focused attention meditation (e.g., “Focus on the sounds and noises in the surrounding environment”) could potentially be used during exercises performed at intensities below ventilatory threshold (i.e., an index of transition between aerobic and anaerobic metabolism) in order to increase the use of dissociative thoughts and enhance one’s affective state. This hypothesis is based on the assumption that such self-regulatory strategies could distract individuals against fatigue-related sensations and facilitate the reflexive control of working muscles (e.g., walking and cycling; Bigliassi et al., 2017).

It is also important to emphasize that mindfulness-based interventions have the potential to ameliorate anxiety and render a given activity more pleasurable than under normal circumstances through the reallocation of attention toward internal bodily signals (e.g., breathing; Xu et al., 2014). Redirecting attention toward the present moment can naturally prevent past and future events from entering focal awareness (van der Zwan et al., 2015). Thoughts about the past and future might serve primarily to induce rumination (i.e., the process of dwelling on stress) and elicit anxiety, given that only task-related thoughts are necessary to complete the exercise session successfully (Alderman et al., 2016; Bigliassi et al., 2020). Therefore, lack of self-regulatory skills can facilitate daydreaming, increase the occurrence of task-unrelated thoughts, elicit negative emotional responses, and potentially compromise the execution of movements.

**FIGURE 2:**
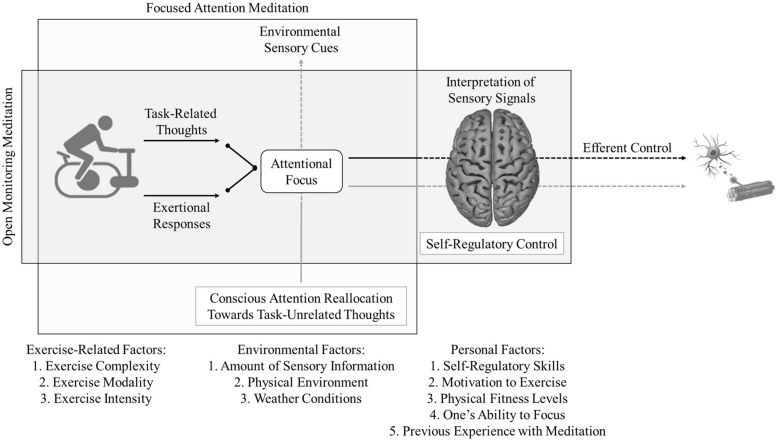
The Self-Regulatory Model of Exercise. Focused attention meditation is proposed to shift attention away from task-related information and facilitate the execution of movements during repetitive tasks performed at intensities below ventilatory threshold. Open monitoring meditation is hypothesized to ameliorate the effects of fatigue and optimize the neural control of working muscles during highly demanding motor tasks performed at and above ventilatory threshold. A series of exercise-related, environmental, and personal factors are included as potential confounds. Gray arrows are representative of a positive influence and black arrows are representative of a negative influence.

Interestingly, meditation techniques could also be used during exercises performed at intensities above ventilatory threshold (for practical examples, see Table 1). This is because meditation has the potential to modulate processing of physical sensations (Sharon et al., 2016) and, consequently, optimize the neural control of the musculature. High-intensity exercises tend to force attention toward internal association (e.g., heavy breathing) and reduce processing of external influences (Hutchinson and Tenenbaum, 2007). In this case, the practice of meditation would function as an effective tool to influence the perception of pain/discomfort and *transform* any negative bodily sensations into motivation to persevere and enhance exercise performance (i.e., assuming the role of an associative strategy; Bigliassi et al., 2016). In a recent study conducted in our laboratory, we have identified that certain types of meditation can be used during moderate-intensity physical tasks performed on a cycle ergometer in order to facilitate handling of undesired thoughts, assuage fatigue, and enhance affective responses^1^. Accordingly, different meditation techniques can be used for different metabolic demands and exercise modalities in order to reduce perceived exertion and optimize the execution of movements (Figure 2). During moderate and high-intensity bouts of physical activity, it appears reasonable to assume that open monitoring meditation may elicit the most positive psychological and psychophysical responses. Exercises performed at intensities above ventilatory threshold leave only a limited capacity for the human brain to process task-irrelevant information (Brick et al., 2014), and, therefore, focused attention meditation might not be particularly potent in such instances. Conversely, open monitoring meditation could potentially imbue individuals with the ability to *decide* what to do with the negative bodily sensations that are commonly experienced during high-intensity exercises (e.g., “If you feel something, simply accept and embrace it. You don’t have to control it but remember that it is your decision how this feeling will affect your performance”).

### Testing the Theoretical Model

It is very important to emphasize that the putative mechanisms proposed in this review article were developed in line with the parallel processing model (Rejeski, 1985), and, therefore, need to be tested through the use of experimental protocols to check its validity and applicability. It is also necessary for future studies in this area of scientific enquiry to clearly describe their methods and procedures in order to allow researchers to confirm/refute the theoretical framework proposed in this article. Thirdly, it is worth noting that a wide variety of factors have the propensity to moderate/mediate the effects of meditation on exercise (Figure 2; Alderman et al., 2016). Exercise-related factors such as complexity (e.g., running on a motorized treadmill and cycling outdoors) and modality (e.g., rowing and walking) need to be taken into consideration while testing the theoretical model proposed herein. For example, focused attention meditation might not be recommended during the execution of complex exercise tasks such as cycling outdoors. This is because of the extensive number of environmental sensory signals that needs to be monitored during the physical task and the safety issues associated with this exercise mode.

There are also a number of environmental and personal factors that require careful consideration while testing this model. One’s ability to focus and experience with the practice of meditation are common examples of personal factors that could potentially influence the effects of meditation on exercise (Figure 2; de Bruin et al., 2017; Kang et al., 2017; Edwards and Loprinzi, 2018). In the present model, focused attention meditation is proposed to have a substantial impact upon the reallocation of attentional focus outwardly, preventing task-relevant signals (e.g., distance covered) from entering focal awareness (Rejeski, 1985). Therefore, processing of internal sensory cues could be down-regulated when individuals execute movements in combination with this type of meditation. Such neuropsychological mechanisms are hypothesized to induce a series of domino reactions (e.g., efficient control of the musculature) that would ultimately delay the disruption of task performance (Bigliassi et al., 2018). It is important to emphasize that task-related thoughts are naturally more relevant than external sensory influences (Rejeski, 1985), and, thus, it is only a matter of time until fatigue-related symptoms overcome the protective effects of focused attention meditation. It is also worth noting that focused attention meditation can be used to guide attention toward internal bodily sensations (e.g., breathing) during light-intensity exercises in order to prevent memories from the past and thoughts about the future from entering focal awareness. This strategy is primarily intended to facilitate emotional reappraisal and elicit a more positive affective state (Ainsworth et al., 2013; Lacaille et al., 2018).

Conversely, open monitoring meditation is hypothesized to enhance self-regulatory control and temper the interpretation of interoceptive signals. In such instances, individuals continue to experience the same physical sensations, thoughts, and emotions (i.e., attentional focus is not redirected), but are taught to perceive such signals differently (Lippelt et al., 2014; Sharon et al., 2016). In the *Self-Regulatory Model of Exercise* proposed in this article, the authors theorize that open monitoring meditation has the potential to prevent negative bodily sensations and emotions from interacting with the activity of the central motor command (i.e., a prophylactic effect). Therefore, the neural control of working muscles could be shielded, to a certain degree, through the use of appropriate meditation techniques (Table 1)^2^.

## Practical Implications

In this section, we provide a series of recommendations on how to use certain types of meditation as a means by which to facilitate emotional reappraisal and exercise re-engagement. We also offer a series of specific orientations for physically active individuals to use meditation-based interventions in order to optimize execution of movements during exercises performed at and above ventilatory threshold. Exercise professionals must bear in mind that the recommendations provided herein still need to be tested in a systematic manner by the scientific community. It is also important to emphasize that a wide range of personal, environmental, and situational factors are likely to moderate the effects of meditation on exercise. Finally, it is worth noting that certain types of meditation might elicit a series of detrimental effects on psychological responses and exercise performance when used inappropriately. Accordingly, it is important to consider the potential influence of exercise-related parameters (e.g., modality, complexity, and duration) when administering certain meditation techniques.

Emotional reappraisal appears to be highly influenced by meditation-based interventions that encompass aspects of gratitude, compassion, and facilitate perception of the present moment (Davis and Hayes, 2011; Garland et al., 2015). Furthermore, such interventions are most effective when administered longitudinally for a period of approximately 6–12 weeks (e.g., Desbordes et al., 2012; Jay et al., 2016). Accordingly, individuals who struggle to re-engage in physical activity programs might attempt to initiate the practice of meditation in order to forge a more solid foundation on which physical activity behavior can be altered. The interventions can also be administered through the use of smartphone applications such as The Mindfulness App, Headspace, and Calm. Recent evidence indicates that such applications are generally effective, inexpensive, and relatively easy to use (for details, see van Emmerik et al., 2018). Moreover, the use of meditation-based mobile applications makes it widely available, allows the practice in various situations, and provides numerous options to consider, which naturally prevents repetitiveness and boredom.

Meditation-based interventions can also be used prior to commencement of an exercise session as a means to regulate one’s arousal state. In such instances, exercise professionals are encouraged to use self-regulation strategies in order to down-modulate perceived activation before the warm-up phase. This approach might be particularly useful when exercisers are feeling stressed or anxious about other aspects of their lives. Accordingly, the use of meditation can be recommended as a strategy to reduce ruminative thinking and muscle tension (Smeets et al., 2014). This can be achieved through the use of mindfulness-based interventions where exercisers are asked to sit motionless for a period of ∼5–10 min and pay attention to any physical sensations, emotions, or environmental sensory stimuli (e.g., auditory signals) in a non-judgmental way. Exercisers may also be asked to perceive any kind of muscle tension in their neck, facial muscles, and lower back, and attempt to relax this musculature. During the final moments of the practice, exercisers might experience improved attentional control and more stable levels of psychophysiological arousal (cf. Birrer and Morgan, 2010; Trautwein et al., 2019).

During execution of movements performed at moderate and high intensities, individuals tend to experience a series of fatigue-related sensations, negative affective responses, and deterioration of motor performance. In this case, audio-guided meditation interventions can be used during the exercise session to facilitate processing of task-related information and change the way individuals perceive their bodily sensations (e.g., “Gently bring your attention to the sensations of discomfort. Your body knows how to fight against the pain you feel. Embrace any pain or discomfort that you are experiencing”)^3^. Certain types of meditation (e.g., body scan, gratitude, and Zen styles) have the potential to imbue exercisers with the ability to counteract the detrimental effects of fatigue and process internal sensory cues with greater acceptance during highly demanding motor tasks (cf. Hilton et al., 2017). This can be achieved when individuals are constantly reminded that the physical sensations they experience during exercise are simply the result of interpretative processes taking place in their brains (e.g., “Although you might experience some discomfort in your legs and chest, these sensations are only interpretations of your brain. It is your decision how these signals will be translated into any kind of discomfort,” “Notice how your legs move up and down during the exercise. It is your decision if they are going to continue moving like this”).

Finally, meditation-based interventions might be particularly effective during the post-exercise phase as means by which to expedite psychophysiological recovery and reduce the likelihood of overtraining. Insufficient recovery from strenuous physical tasks can lead to a myriad of detrimental effects on psychological and physiological responses (Meeusen et al., 2007; Coote, 2010). Such responses are commonly associated with residual effects that linger from one exercise session to another. Consequently, task performance in subsequent training sessions tends to be somehow affected by previous bouts of exercise (Piacentini and Meeusen, 2015). The recovery processes that follow strenuous physical tasks may also be influenced by meditation-based interventions employed immediately after completion of the exercise (Kudlackova et al., 2013). Compelling evidence indicates that when individuals experience positive affective responses during the post-exercise recovery phase, they also tend to exhibit lower levels of cortisol when compared to normal conditions (Karageorghis et al., 2018). Therefore, strategies that aim to assuage the physiological stress imposed by physical tasks and elicit a more positive affective response during the post-exercise recovery phase might be relevant to exercise professionals. In such circumstances, mindfulness-based intervention might be used as valuable tools to make individuals more aware of their physical sensations and emotions during the post-exercise recovery phase. This strategy may also serve to optimize self-control (e.g., “Notice how your abdomen is continuously moving. There is nothing you have to do to control it. Your body knows how to control itself”) and return the organism to a homeostatic state through a sequence of physiological adjustments (see Kudlackova et al., 2013; Romero et al., 2017).

## Recommendations for Future Studies

Mindfulness-based interventions and other forms of meditation can be applied very briefly prior to commencement of any exercise session, during the execution of movements, and even after exercise completion (Table 1). This might have the potential to facilitate mental preparation for the session ahead, assuage negative bodily sensations (e.g., muscle ache) during the practice, and even expedite psychophysiological recovery (Bernier et al., 2009). In Table 1 a series of meditation techniques are recommended on the basis of their applicability and functionality (aerobic and anaerobic demands). Practical examples are also provided as a means by which to facilitate the implementation of meditation prior to, during, and immediately after the exercise routines.

We have also provided a series of recommendations for future studies. Firstly, researchers need to guarantee that participants were executing the task successfully. This is mainly because some individuals might require long periods of exposure to be able to practice meditation during highly demanding motor tasks. In such instances, manipulation checks can be conducted through the use of open questions and semi-structured interviews to gauge the participants’ perceptions of the meditation routine. It is also recommended that a familiarization process is conducted during the pre-experimental phase of the study whenever participants are unfamiliar with the practice of meditation. Taking the present models into consideration (Figures 1, 2), researchers should monitor and report any environmental, personal, and exercise-related factors that might bear an influence on the effects of meditation. Finally, it is of paramount importance to standardize the experimental manipulations, so the studies are comparable to a certain degree (for details, see Bigliassi et al., 2020). Participants should be exposed to the same experimental protocol and the scripts used for each session should be provided as supplementary files whenever possible.

## Conclusion

Self-regulation strategies such as meditation appear to have a potent effect on a wide range of physical, psychological, and behavioral outcomes (Davidson et al., 2003; Alderman et al., 2016). Similarly, physical exercise has been recurrently proven to be one of the most efficient interventions to counteract the detrimental effects of depression, anxiety, and stress (Pedersen and Saltin, 2015). Therefore, it appears reasonable to assume that exercise and meditation are perfect “bed fellows” (de Bruin et al., 2017). It is hypothesized that the psychological, physiological, and psychophysiological benefits of exercise could be maximized when performed in combination with meditation routines. Also, there is the possibility of an additional effect where meditation practices might have the potential to facilitate execution of movements and enhance task performance. This is primarily based on the fact that meditation can rearrange the electrical frequency in the cerebral cortex (Berkovich-Ohana et al., 2013), influence the communication across brain regions (Brewer et al., 2011; Bigliassi et al., 2020), and facilitate emotional reappraisal (Lacaille et al., 2018). Such mechanism could lead to a series of subsequent effects on psychophysical (e.g., perceived exertion) and psychobiological (e.g., cortisol levels) parameters during the exercise session and, potentially, during the post-exercise recovery phase.

## Author Contributions

MB and RB reviewed the literature, developed the theoretical framework, and wrote the manuscript.

## Conflict of Interest

The authors declare that the research was conducted in the absence of any commercial or financial relationships that could be construed as a potential conflict of interest.

## Footnotes

1 Bigliassi, M., Ocubaro, A. M., Lima-Silva, A. E., Buzzachera, C. F., and Bertuzzi, R. (submitted for publication). Effects of meditation on psychological and psychophysiological responses during moderate-intensity exercise: an EEG study.

2 Bigliassi, M., Ocubaro, A. M., Lima-Silva, A. E., Buzzachera, C. F., and Bertuzzi, R. (submitted for publication). Effects of meditation on psychological and psychophysiological responses during moderate-intensity exercise: an EEG study.

3 Bigliassi, M., Ocubaro, A. M., Lima-Silva, A. E., Buzzachera, C. F., and Bertuzzi, R. (submitted for publication). Effects of meditation on psychological and psychophysiological responses during moderate-intensity exercise: an EEG study.
